# Predictors of unacceptable pain with and without low inflammation over 5 years in early rheumatoid arthritis—an inception cohort study

**DOI:** 10.1186/s13075-021-02550-7

**Published:** 2021-06-14

**Authors:** Anna Eberhard, Stefan Bergman, Thomas Mandl, Tor Olofsson, Maria Rydholm, Lennart Jacobsson, Carl Turesson

**Affiliations:** 1 Rheumatology, Department of Clinical Sciences, Malmö, Lund University, Jan Waldenströms gata 1b, 214 28 Malmö, Sweden; 2 Rheumatology, Department of Clinical Sciences, Lund, Lund University, Lund, Sweden; 3 Department of Public Health and Community Medicine, Institute of Medicine, Sahlgrenska Academy, University of Gothenburg, Gothenburg, Sweden; 4 Department of Rheumatology, Skåne University Hospital, Malmö, Sweden; 5 Department of Rheumatology and Inflammation Research, Sahlgrenska Academy, Gothenburg, Sweden

**Keywords:** Rheumatoid arthritis, Pain, Predictor, Non-inflammatory pain

## Abstract

**Objectives:**

Pain is a major symptom in patients with rheumatoid arthritis (RA). In early RA, pain is usually due to synovitis, but can also persist despite effective anti-inflammatory treatment. The objective of this study was to investigate the pain course over time and predictors of unacceptable pain and unacceptable pain with low inflammation, in patients with early RA.

**Methods:**

An inception cohort of 232 patients with early RA, recruited in 1995–2005, was followed in a structured programme for 5 years. Pain was assessed using a visual analogue scale (VAS; 0–100). Unacceptable pain was defined as VAS pain > 40 based on the patient acceptable symptom state (PASS) and low inflammation as CRP < 10 mg/l. Baseline predictors of unacceptable pain were evaluated using logistic regression analysis.

**Results:**

Pain improved significantly during the first 6 months, but then remained basically unchanged. Thirty-four per cent of the patients had unacceptable pain 5 years after inclusion. Baseline predictors of unacceptable pain after 5 years were lower swollen joint counts [odds ratio (OR) 0.71 per standard deviation (95% confidence interval (CI) 0.51–0.99)] and higher VAS for pain and global assessment of disease activity. Unacceptable pain with low inflammation after 5 years was negatively associated with anti-CCP antibodies [OR 0.50 (95% CI 0.22–0.98)].

**Conclusion:**

Over one third of the patients had unacceptable pain 5 years after inclusion. Lower swollen joint count was associated with unacceptable pain at 5 years. The results may be explained by the positive effects of treatment on pain related to inflammation. Non-inflammatory long-lasting pain appears to be a greater problem in anti-CCP-negative patients.

**Supplementary Information:**

The online version contains supplementary material available at 10.1186/s13075-021-02550-7.

## Introduction

Rheumatoid arthritis (RA) is a chronic autoimmune inflammatory disease characterized by inflammation of the joints, resulting in pain, stiffness and destruction of articular bone and cartilage. With more extensive treatment with disease-modifying anti-rheumatic drugs (DMARDs) in recent years, the average disease course, measured using patient-reported outcomes (PROs) [1], clinical status, radiographic scores and laboratory markers of inflammation [2], has greatly improved. Nevertheless, a significant proportion of patients with RA still suffer from pain [3–5], and a subgroup of patients report pain despite apparent inflammatory control [6, 7]. In a longitudinal study of British patients with RA, 65% of those achieving a disease state of low inflammation had persistent pain over time [8].

Painful arthritis is, in part, due to effects of inflammatory cytokines, which activate nociceptors in the synovium [9]. It has been hypothesized that central sensitisation could also contribute to pain in RA, and this is supported by several studies [8, 10, 11]. Such nociplastic pain could thus possibly explain why a subgroup of patients with RA have pain despite inflammation control.

Pain is a debilitating symptom and has been associated with reduced health-related quality of life and increased disability [12, 13], and also with future depressive symptoms and work disability [14–17]. In a systematic literature review, the authors report that pain reduction was the most common goal for patients with RA, expressed by 81% of patients [18]. Improved pain treatment could have many beneficial effects other than reducing pain. Some authors have argued that it is pain, and not disease activity, which drives fatigue, and that interventions to reduce pain might also have beneficial effects on fatigue [19]. Reducing pain could also reduce chronic opioid use in RA, which has been shown to increase over time [20]. Furthermore, remaining pain has been associated with more sickness absence [21], and improved pain management might hence also be beneficial for patients’ work ability.

In a recent study, pain trajectories in RA patients were investigated in 3 different cohorts. A low pain trajectory was identified, with patients who had low pain during the whole follow-up time, but only in the cohort of early RA patients, suggesting that early interventions are of importance for pain management [8]. By finding pain predictors at the beginning of the disease, patients with an increased risk of long-lasting pain can be identified, thus making it possible to start early pain-targeted interventions.

The aims of this study were to examine (1) the course of pain and proportion of unacceptable pain during the first 5 years of early RA, (2) predictors of change in pain, and (3) predictors of unacceptable pain, overall and with low inflammatory activity.

## Patients and methods

### Patients

An inception cohort of patients with early RA consisting of 233 patients from the city of Malmö, Sweden, recruited in 1995–2005 with a symptom duration ≤ 12 months at inclusion was investigated [22, 23]. Patients were recruited from the rheumatology outpatient clinic of Malmö University Hospital, the only hospital serving the city, as well as from the 4 rheumatologists in private practice in Malmö. All patients fulfilled the 1987 American College of Rheumatology criteria for RA [24] and were diagnosed by a specialist in rheumatology. The study was approved by the Regional Ethical Review Board for southern Sweden, and all participants gave their written informed consent before inclusion in the study.

### Clinical assessment

Patients were followed in a structured programme with examination and collection of data at inclusion, 6 months and 1, 2 and 5 years. There was no pre-specified protocol for pharmacotherapy—all patients were managed according to standard care. PROs and disease activity measures were collected at every follow-up visit. A visual analogue scale (VAS; range 0–100 mm) was used for assessing pain, as well as the patients’ global assessment of disease activity (PGA). The number of swollen and tender joints (out of 28) was assessed by the same rheumatologist for all patients at all visits. Disability was evaluated using the Swedish validated version of the Stanford Health Assessment Questionnaire (HAQ) [25]. For disease activity measures, the Disease Activity Score in 28 joints (DAS28) was used. Information on ongoing treatment with DMARDs and glucocorticosteroids was obtained through an interview at every visit. Data on treatment with biologic DMARDs were obtained through linkage to the regional biologics register with 95% coverage in the area. Radiographs of hands and feet were performed, and the presence of erosions (present vs absent) was assessed by a radiologist as part of standard clinical practice. Grip force (Newton) was measured using the electronic instrument Grippit (AB Detektor). Grip force values for the dominant hand were obtained as previously described [23], and compared to age- and sex-specific reference values from the literature [26]. Grip force was expressed as a percentage of the expected value.

### Laboratory measures

As markers of ongoing inflammation, the erythrocyte sedimentation rate (ESR) and C-reactive protein (CRP) were measured, using standard methods at the Department of Clinical Chemistry at Malmö University Hospital. At inclusion, all patients were tested for rheumatoid factor (RF) and anti-cyclic citrullinated peptide (anti-CCP) seropositivity, using standard ELISA methods at the immunology laboratories of the University Hospitals in Malmö and Lund. IgM RF was analysed using ELISA, which was calibrated against the World Health Organization RF reference preparation. Anti-CCP antibodies were analysed using the Quanta Lite CCP IgG ELISA (INOVA Diagnostics, USA).

### Outcomes: unacceptable pain with and without low inflammation

Unacceptable pain was defined as VAS pain > 40 mm, based on the patient acceptable symptom state (PASS) [27], which is a validated measure, captured from patient reports, indicating the cut-off level of acceptable pain. The aspect of unacceptable pain despite low inflammation was also assessed, in order to investigate pain patterns indicative of non-inflammatory pain, with low inflammation defined according to the previously used definition of CRP < 10 mg/l [6, 28]. A more strict definition of low inflammation was also included, including those with CRP < 10 mg/l and swollen joint count out of 28 (SJC28) ≤ 1 [6]. High inflammation was defined as CRP ≥ 10 mg/l.

### Statistical analysis

IBM SPSS statistics version 26 was used for statistical analyses. The development of pain over time was assessed using descriptive statistics, and the change in pain between every visit was evaluated using the paired t test. To determine the normality distribution of data, the Shapiro-Wilk test was used. Confidence intervals for the proportion of patients with unacceptable pain were computed using the Wald method with normal approximation. If observed cases were < 5 mid-P exact intervals were used. Potential baseline predictors of unacceptable pain, and of unacceptable pain with low inflammation, were assessed using univariate and multivariate logistic regression analysis. Results were presented as odds ratios (OR) with 95% confidence intervals (CI). Continuous variables were analysed per standard deviation and were tested for linearity to the logit of the dependent variable. As a secondary analysis, predictors of unacceptable pain with high inflammation were also assessed. Since the method for measuring CRP during part of the period was not highly sensitive and did not include any values below 9, CRP values were analysed by groups of CRP < 9 mg/l and the two highest quartiles, with the first group as reference. Variables with a p value < 0.10 were eligible for multivariate analyses and were assessed for bivariate correlations, using Spearman’s rank correlation test. In the case of collinearity (bivariate correlation between covariates with r > 0.3), only the covariate with the strongest association with the outcome variable was included in the multivariate logistic regression model. Sensitivity analyses were also performed with adjustment for year of inclusion and place of practice (university vs private).

Baseline predictors of pain over time were assessed using mixed model analysis, using all VAS pain values at inclusion and follow-ups at 6 months, 1, 2 and 5 years. Differences in pain at baseline by baseline characteristics were estimated as the intercept, based on the regression line. Mean differences in pain over time and differences in change of pain per month were estimated.

## Results

### Patient characteristics

A total of 232 patients with early RA (median symptom duration 7 months (interquartile range: 5–10)) were included in the study. All the patients fulfilled 1987 ACR criteria for RA. Due to missing data for some parameters (i.e., data on RF levels and details on joint tenderness in the feet were not available), the 2010 ACR/EULAR criteria could not be fully evaluated, but at least 88% (204/232) of the patients fulfilled these criteria at inclusion. The 5-year follow-up was attended by 179 patients. Patient characteristics at inclusion, 6 months and 1, 2 and 5 years are shown in Table 1. The majority of patients was treated with methotrexate. During the 5-year period, 17% were at some point treated with a biologic DMARD.

**Table 1:** Patient characteristics in patients with early RA at inclusion and at follow-up visits

Characteristic	Inclusion	6 months	1 year	2 years	5 years
N	232	212	219	208	179
Sex, female, n (%)	169 (70.3)	150 (70.8)	155 (70.8)	146 (70.2)	127 (70.9)
Age, years, mean (SD)	60.5 (14.6)	60.4 (14.5)	60.6 (14.6)	61.5 (14.9)	63.7 (14.6)
Symptom duration at inclusion, months	7.0 (5.0–10.0)	7.0 (5.0–10.0)	7.0 (5.0–10.0)	7.0 (5.0–10.0)	7.0 (5.0–10.0)
RF positive at inclusion, n (%)	143 (61.6)	127 (59.9)	135 (61.6)	125 (60.1)	115 (64.2)
Anti-CCP positive at inclusion, n/N (%)	116/202 (57.4)	106/185 (57.3)	109/189 (57.7)	102/180 (56.7)	91/155 (58.7)
Prednisolone, n (%)	90 (38.8)	77 (36.3)	69 (31.5)	63 (30.3)	52 (29.1)
Methotrexate, n (%)	124 (53.4)	125 (59.0)	137 (62.6)	128 (61.5)	110 (61.5)
Biologic DMARD, n (%)	0 (0)	5 (2.4)	12 (5.5)	17 (8.2)	32 (17.9)
> 1 csDMARD, n (%)	4 (1.72)	14 (6.6)	14 (6.4)	20 (9.6)	16 (8.9)
No DMARD, n (%)	41 (17.7)	28 (13.2)	28 (12.8)	36 (17.3)	42 (23.5)
Erosion, n (%)	35 (15.1)	NA	55 (25.1)	68 (32.7)	70 (39.1)
Body Mass Index, mean (SD)	25.4 (4.2)^a^	NA	NA	25.9 (4.5)^b^	NA
Current smoking, n/N (%)	57/165 (34.5)	NA	NA	NA	NA
Grip force, % of expected, mean (SD)	39.8 (25.7)^c^	48.9 (27.1)^d^	51.8 (27.3)^e^	54.1 (28.5)^f^	56.9 (30.3)^g^
VAS pain, mean (SD)	41.2 (26.8)	32.3 (26.2)	30.1 (24.1)	32.1 (27.0)	30.3 (23.8)
DAS28, mean (SD)	4.6 (1.4)	3.8 (1.4)	3.7 (1.4)	3.6 (1.4)	3.6 (1.4)
SJC28	7.0 (5.0–11.0)	4.5 (2.0–7.0)	4.0 (2.0–7.0)	4.0 (2.0–7.0)	4.0 (2.0–7.0)
TJC28	4.0 (1.0–9.0)	2.0 (0–6.0)	2.0 (0–5.0)	1.0 (0–4.0)	1.0 (0–3.0)
HAQ	0.8 (0.4–1.3)	0.5 (0.1–0.9)	0 (0–1.0)	0.5 (0–1.0)	0.8 (0.1–1.1)
CRP (mg/l)	9.0 (< 9–26.8)	< 9 (< 9–11.0)	< 9 (< 9–10.0)	< 9 (< 9–11.0)	< 9 (< 9–9.3)
CRP > 9 mg/l, n (%)	121 (52.2)	61 (28.8)	58 (26.5)	62 (29.8)	44 (24.7)
ESR (mm/h)	20.5 (10.0–43.0)	14.0 (8.0–30.0)	15.0 (8.0–27.0)	15.0 (8.0–26.3)	15.0 (9.0–24.0)
VAS PGA, mean (SD)	43.3 (26.7)	33.3 (25.2)	30.6 (23.9)	33.6 (26.5)	34.5 (24.7)

Legend: Values are median (interquartile range) unless otherwise indicated. ^a^Data for body mass index in 162 cases. ^b^Data in 139 cases. ^c^Data for grip force in 200 cases. ^d^ Data in 180 cases. ^e^Data in 198 cases. ^f^Data in 200 cases. ^g^Data in 173 cases

*SD* standard deviation, *RF* rheumatoid factor, *Anti-CCP* anti-cyclic citrullinated peptide, *DMARD* disease-modifying anti-rheumatic drug, *csDMARD* conventional synthetic disease-modifying anti-rheumatic drug, *NA* not available, *VAS* visual analogue scale, *DAS28* disease activity score in 28 joints, *SJC28* swollen joint count in 28 joints, *TJC28* tender joint count in 28 joints, *HAQ* health assessment questionnaire, *CRP* C-reactive protein, *ESR* erythrocyte sedimentation rate, *PGA* patient global assessment

### Pain over time

**Fig. 1:**
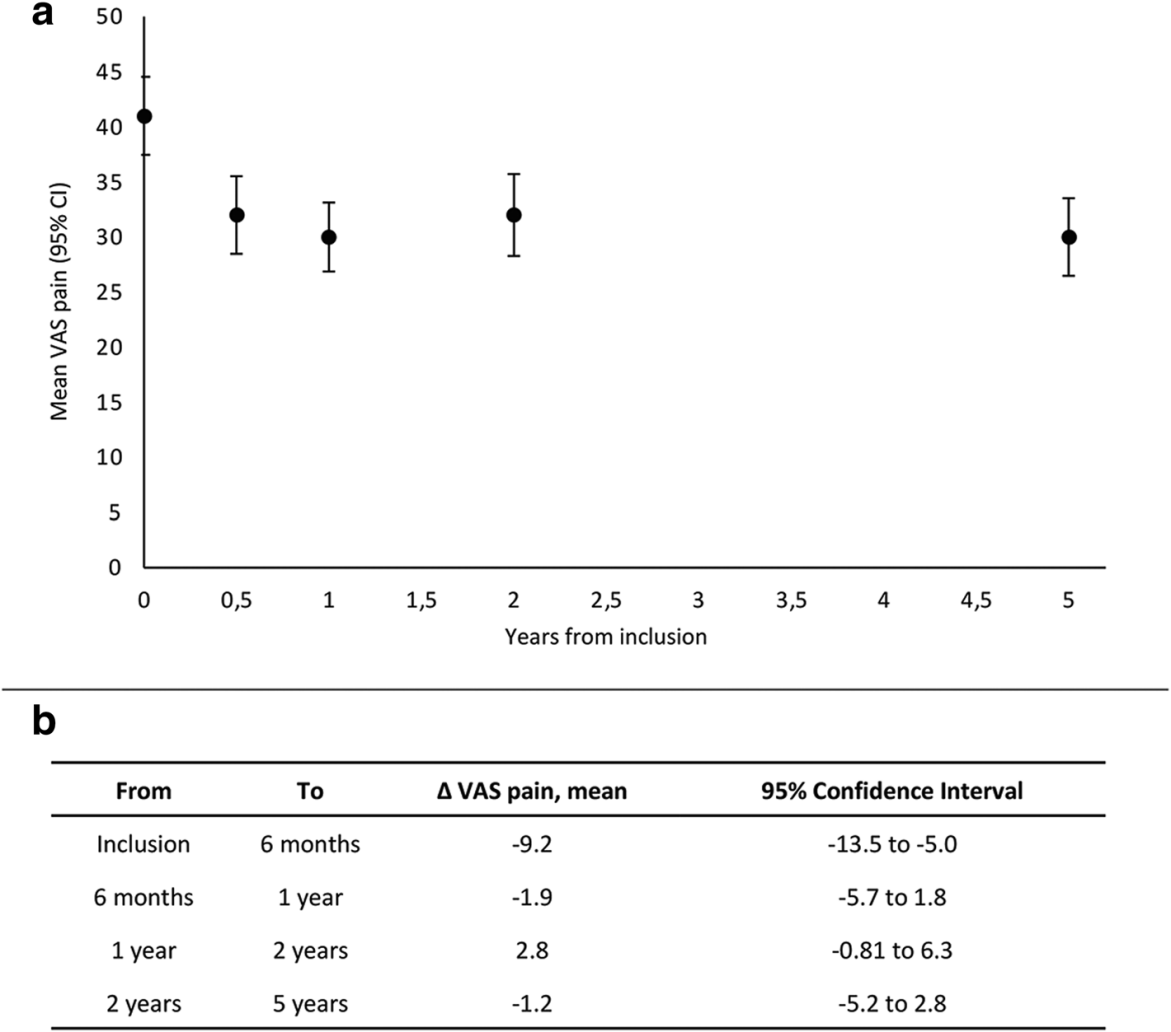
**a** Mean VAS pain from inclusion to 5 years in patients with early RA. Legend: Mean VAS pain over time, with 95% confidence intervals. There was a reduction in mean VAS pain from inclusion to 6 months. Mean VAS pain was thereafter more or less unchanged over time. **b** Mean change in pain between every follow-up visit. Legend: Paired samples t test. VAS pain decreased significantly from inclusion to 6 months. After 6 months there was no significant change in pain between the follow-up visits. VAS: visual analogue scale

The mean VAS pain was 41.2 at inclusion and decreased significantly to 32.3 at the 6-month visit, but then remained more or less unchanged during the rest of the follow-up period (Fig. 1A). The mean change in VAS pain from inclusion to 6 months was − 9.2 (p < 0.001). After 6 months, there was no significant change in pain between the follow-up visits (Fig. 1B).

**Fig. 2:**
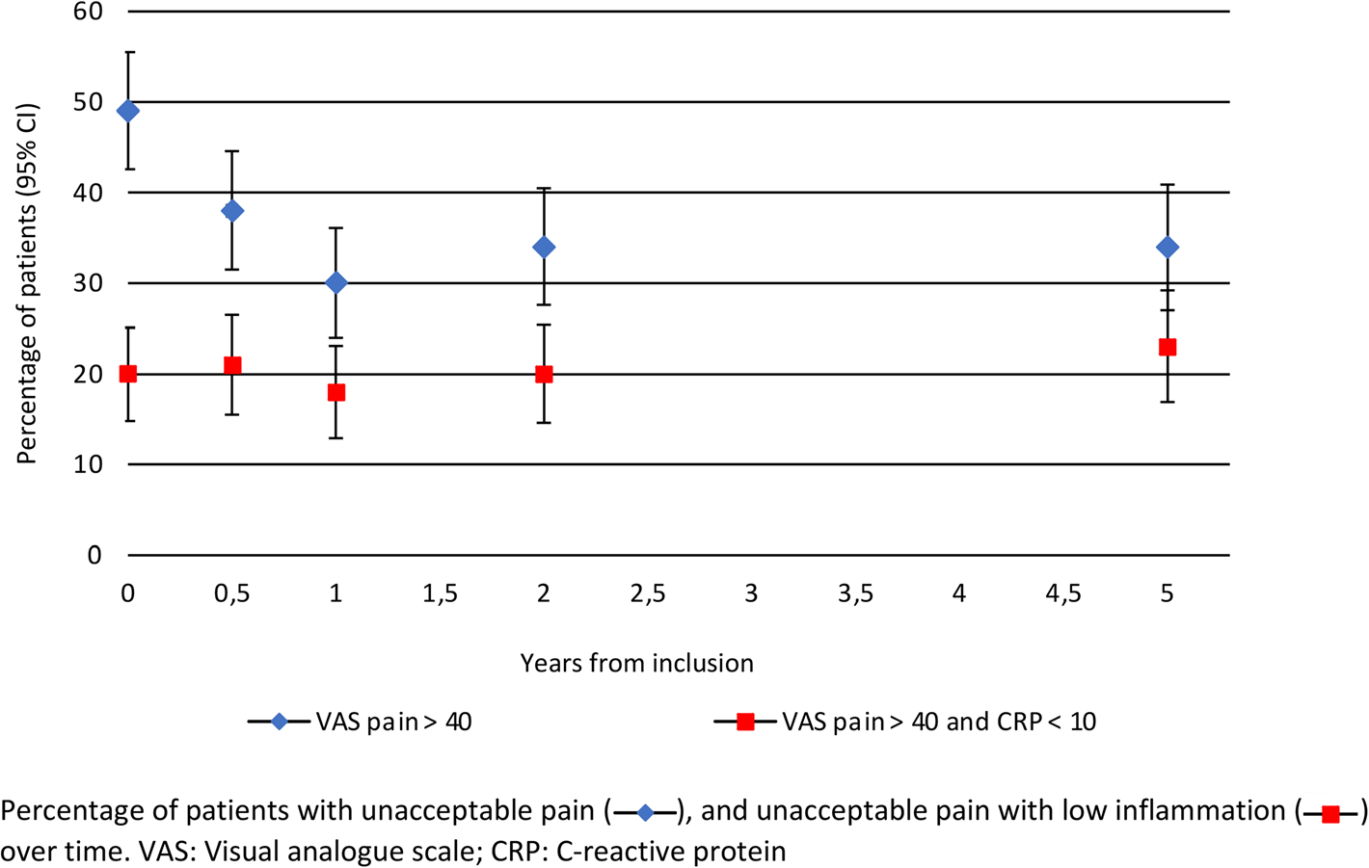
Percentage of patients with unacceptable pain over time, overall and in patients with low inflammation, in early RA

The proportion of patients with unacceptable pain at inclusion was 49.1% and decreased to 30.1% during the first year. After that, the fraction of patients with unacceptable pain was essentially unchanged over time (Fig. 2). At inclusion, 20.2% had unacceptable pain with low inflammation and 2.2% had unacceptable pain with the strict definition of low inflammation. The proportion of patients with unacceptable pain and low inflammation, as well as with the strict definition of low inflammation, did not change significantly during the 5-year follow-up (Fig. 2).

### Predictors of unacceptable pain—univariate and multivariate analyses

Baseline predictors of unacceptable pain at 6 months and 1, 2 and 5 years from inclusion were identified. Baseline treatment with methotrexate was similar in patients with and without unacceptable pain at each of the follow-up visits (Additional file 1). There were 66 patients (30.1%) with unacceptable pain at 1 year and 71 (34.1%) at 2 years. In univariate analyses, higher baseline VAS pain, HAQ and PGA scores were associated with unacceptable pain at 1 and 2 years (Table 2). Unacceptable pain at 2 years was also associated with female sex and lower age at inclusion, and there was a negative association with grip force (Table 2). In multivariate analysis of baseline predictors of unacceptable pain at 2 years, there were significant associations for female sex [OR 2.57 (95% CI 1.27–5.33)] and for VAS pain [OR 1.56 per SD (95% CI 1.14–2.14)] (Table 3).

**Table 2:** Baseline predictors of unacceptable pain in early RA – 6 months, 1, 2 and 5 years after diagnosis

	6 months	1 year	2 years	5 years
Variable	Odds ratio (95% CI)	Odds ratio (95% CI)	Odds ratio (95% CI)	Odds ratio (95% CI)
Female sex	0.94 (0.51–1.73)	0.93 (0.49–1.75)	**2.48 (1.24 –5.0)**	1.24 (0.62–2.47)
RF seropositivity	1.54 (0.86–2.74)	1.03 (0.57–1.87)	1.35 (0.75–2.45)	0.88 (0.46–1.67)
Anti-CCP seropositivity	1.20 (0.65–2.19)	1.63 (0.87–3.07)	1.09 (0.59–2.03)	0.77 (0.39–1.49)
Erosion	0.53 (0.22–1.24)	0.55 (0.23–1.34)	0.41 (0.16–1.06)	0.62 (0.26–1.49)
Age	0.86 (0.65–1.14)	0.82 (0.61–1.08)	**0.71 (0.54–0.95)**	1.09 (0.80–1.49)
Symptom duration	1.11 (0.83–1.47)	1.22 (0.91–1.64)	1.03 (0.77–1.37)	1.28 (0.93–1.76)
Body Mass Index	1.17 (0.85–1.62)	1.04 (0.74–1.45)	0.98 (0.70–1.38)	0.95 (0.64–1.40)
Current smoking	1.24 (0.62–2.46)	1.53 (0.76–3.08)	0.72 (0.34–1.52)	1.39 (0.64–3.02)
Grip force	0.83 (0.61–1.14)	0.73 (0.52–1.04)	**0.71 (0.51–0.99)**	0.90 (0.65–1.24)
VAS pain	**1.50 (1.12–1.99)**	**1.69 (1.25–2.30)**	**1.55 (1.15–2.09)**	**1.40 (1.02–1.91)**
DAS28	**1.44 (1.08–1.93)**	1.26 (0.94–1.69)	1.25 (0.94–1.67)	1.03 (0.76–1.41)
SJC28	1.05 (0.80–1.38)	0.83 (0.62–1.12)	0.94 (0.71–1.25)	**0.71 (0.51–0.99)**
TJC28	1.24 (0.94–1.62)	1.20 (0.91–1.59)	1.11 (0.85–1.46)	0.98 (0.71–1.35)
HAQ	1.31 (0.99–1.74)	**1.46 (1.10–1.95)**	**1.57 (1.18–2.11)**	1.01 (0.74–1.37)
CRP < 9 mg/l	1.00 (reference)	1.00 (reference)	1.00 (reference)	1.00 (reference)
CRP 9–27.4 mg/l	0.77 (0.39–1.51)	1.09 (0.55–2.17)	0.64 (0.31–1.31)	1.16 (0.57–2.38)
CRP 27.5–174 mg/l	0.82 (0.41–1.63)	0.79 (0.38–1.64)	0.96 (0.48–1.94)	0.60 (0.27–1.36)
ESR (mm/h)	1.17 (0.89–1.54)	0.99 (0.74–1.32)	1.14 (0.86–1.51)	0.79 (0.57–1.09)
VAS PGA	**1.59 (1.19–2.12)**	**1.44 (1.07–1.94)**	**1.52 (1.13–2.05)**	**1.60 (1.16–2.21)**

Legend: Univariate logistic regression analysis. Odds ratios are calculated per standard deviation for continuous variables. Values in bold indicate statistical significance with p values < 0.05. Unacceptable pain: VAS pain > 40. *CI* confidence interval, *RF* rheumatoid factor, *Anti-CCP* anti-cyclic citrullinated peptide, *VAS* visual analogue scale, *DAS28* disease activity score in 28 joints, *SJC28* swollen joint count in 28 joints, *TJC28* tender joint count in 28 joints, *HAQ* health assessment questionnaire, *CRP* C-reactive protein, *ESR* erythrocyte sedimentation rate, *PGA* patient global assessment

**Table 3:** Baseline predictors of unacceptable pain in early RA, multivariate analysis

Variable	Odds ratio	95% CI	P value
**2 years after inclusion**			
VAS pain	1.56	1.14–2.14	< 0.01
Female sex	2.57	1.27–5.33	0.01
Age	0.80	0.60–1-07	0.13
Erosion	0.54	0.20–1.44	0.22
**5 years after inclusion**			
PGA	1.78	1.26–2.52	< 0.01
SJC28	0.61	0.42–0.89	0.01

Legend: Multivariate logistic regression analysis. Odds ratios are calculated per standard deviation for continuous variables. Unacceptable pain: VAS pain > 40. *CI* confidence interval, *VAS* visual analogue scale, *PGA* patient global assessment, *SJC28* swollen joint count in 28 joint, *PGA* patient global assessment

Five years after inclusion, 61 patients (34.1%) still had unacceptable pain. In univariate analysis, baseline predictors of unacceptable pain at 5 years were, again, higher VAS pain and VAS PGA (Table 2). There was also a negative association with the SJC at inclusion. Unacceptable pain at 5 years was not associated with female sex or baseline HAQ. In multivariate logistic regression analysis including baseline VAS PGA and SJC, both remained significant predictors of unacceptable pain at 5 years [adjusted ORs 1.78 (95% CI 1.26–2.52) per SD for VAS PGA and 0.61 (95% CI 0.42–0.89) per SD for SJC]. Adjustment for year of inclusion and place of practice did not have a major impact on the results (additional file 2).

### Predictors of unacceptable pain with low and high inflammation—univariate and multivariate analyses

To investigate pain patterns indicative of a non-inflammatory mechanism, baseline predictors of unacceptable pain with low inflammation were also identified. Patient characteristics at baseline in patients with unacceptable pain plus low inflammation at 6 months and 1, 2 and 5 years are shown in Additional file 3. One year after inclusion 40 patients (18.3%) had unacceptable pain with low inflammation. In univariate analysis, baseline predictors of unacceptable pain with low inflammation at 1 year were higher VAS pain, lower age and lower ESR (Table 4). There were negative associations between CRP in the highest quartile at baseline and unacceptable pain with low inflammation (Table 4). In addition, unacceptable pain with low inflammation at 2 years was associated with female sex at inclusion, and at the 6-month visit there was a negative association with baseline erosion. In multivariate analysis, lower age was associated with unacceptable pain with low inflammation at 2 years, with a similar trend at 1 year (Table 5).

**Table 4:** Baseline predictors of unacceptable pain and low inflammation—6 months and 1, 2 and 5 years after diagnosis

	6 months	1 year	2 years	5 years
Variable	Odds ratio (95% CI)	Odds ratio (95% CI)	Odds ratio (95% CI)	Odds ratio (95% CI)
Female sex	1.15 (0.55–2.41)	0.83 (0.40–1.74)	**2.94 (1.17–7.41)**	1.87 (0.80–4.40)
RF seropositivity	0.72 (0.37–1.39)	0.72 (0.36–1.44)	0.81 (0.41–1.63)	0.54 (0.26–1.09)
Anti-CCP seropositivity	0.79 (0.39–1.60)	0.94 (0.45–1.94)	0.62 (0.29–1.31)	**0.50 (0.22–0.98)**
Erosion	**0.11 (0.01–0.81)**	0.55 (0.18–1.66)	0.24 (0.56–1.07)	0.62 (0.22–1.72)
Age	0.79 (0.58–1.10)	**0.62 (0.45–0.86)**	**0.61 (0.44–0.85)**	0.97 (0.68–1.38)
Symptom duration	1.21 (0.86–1.69)	1.37 (0.96–1.96)	0.98 (0.69–1.38)	1.19 (0.83–1.71)
Body mass index	1.28 (0.89–1.86)	0.92 (0.61–1.38)	0.95 (0.64–1.45)	0.98 (0.63–1.52)
Current smoking	0.49 (0.20–1.22)	1.07 (0.46–2.53)	0.42 (0.15–1.20)	0.77 (0.31–1.94)
Grip force	0.97 (0.68–1.37)	0.88 (0.61–1.29)	0.82 (0.56–1.21)	0.88 (0.61–1.28)
VAS pain	1.28 (0.92–1.78)	**1.42 (1.00–2.01)**	1.28 (0.91–1.80)	1.34 (0.94–1.92)
DAS28	1.00 (0.72–1.40)	0.96 (0.68–1.34)	0.98 (0.70–1.37)	1.00 (0.70–1.42)
SJC28	0.88 (0.63–1.24)	0.80 (0.55–1.14)	0.89 (0.63–1.25)	0.70 (0.47–1.03)
TJC28	1.23 (0.90–1.67)	1.18 (0.85–1.63)	1.14 (0.83–1.57)	1.04 (0.72–1.50)
HAQ	0.99 (0.71–1.38)	1.06 (0.76–1.49)	1.13 (0.81–1.57)	1.01 (0.72–1.43)
CRP < 9 mg/l	1.00 (reference)	1.00 (reference)	1.00 (reference)	1.00 (reference)
CRP 9–27.4 mg/l	0.50 (0.23–1.13)	0.65 (0.30–1.43)	0.66 (0.29–1.49)	1.37 (0.63–3.0)
CRP 27.5–174 mg/l	**0.25 (0.09–0.68)**	**0.05 (0.01–0.40)**	**0.33 (0.12–0.92)**	0.41 (0.14–1.18)
ESR (mm/h)	0.72 (0.49–1.05)	**0.59 (0.38–0.93)**	0.66 (0.44–1.01)	0.74 (0.50–1.11)
VAS PGA	1.14 (0.82–1.59)	1.12 (0.80–1.58)	1.24 (0.88–1.74)	1.39 (0.98–2.00)

Legend: Univariate logistic regression analysis. Odds ratios are calculated per standard deviation for continuous variables. Unacceptable pain: VAS pain > 40. Low inflammation: CRP < 10 mg/l. Values in bold indicate statistical significance with p values < 0.05. *CI* confidence interval, *RF* rheumatoid factor, *Anti-CCP* anti-cyclic citrullinated peptide, *VAS* visual analogue scale, *DAS28* disease activity score in 28 joints, *SJC28* swollen joint count in 28 joints, *TJC28* tender joint count in 28 joints, *HAQ* health assessment questionnaire, *CRP* C-reactive protein, *ESR* erythrocyte sedimentation rate, *PGA* patient global assessment.

**Table 5:** Baseline predictors of unacceptable pain with low inflammation in early RA, multivariate analysis

Variable	Odds ratio	95% CI	P value
1 year after inclusion			
VAS pain	1.62	1.11–2.39	0.01
ESR	0.54	0.33–0.88	0.01
Age	0.72	0.51–1.01	0.06
2 years after inclusion			
Female sex	2.41	0.93–6.23	0.07
Age	0.71	0.50–0.99	0.04
Erosion	0.30	0.07–1.38	0.12
CRP < 9 mg/l	1.00 (reference)	-	-
CRP 9–27.4 mg/l	0.91	0.39–2.17	0.84
CRP ≥ 27.5 mg/l	0.49	0.17–1.42	0.19
5 years after inlcusion			
Anti-CCP seropositivity	0.43	0.20–0.95	0.04
SJC28	0.63	0.39–1.00	0.05
PGA	1.51	1.01–2.25	0.04

Legend: Multivariate logistic regression analysis. Odds ratios are calculated per standard deviation for continuous variables. Unacceptable pain: VAS pain > 40. Low inflammation: CRP < 10 mg/l. *CI* confidence interval, *VAS* visual analogue scale, *ESR* erythrocyte sedimentation rate, *CRP* C-reactive protein, *Anti-CCP* anti-cyclic citrullinated peptide, *SJC28* swollen joint count in 28 joint, *PGA* patient global assessment

At the 5-year follow-up, 40 patients (22.5%) had unacceptable pain with low inflammation. Anti-CCP negativity was the only significant baseline predictor of this state in both univariate and multivariate analysis (Tables 4 and 5). In multivariate analysis, a higher baseline VAS PGA was also predictive of unacceptable pain with low inflammation, with a similar trend for lower SJC at baseline (Table 5). Adjustment for year of inclusion and practice did not have a major impact on the results (additional file 4). Unfortunately, it was not possible to perform analyses of predictors of unacceptable pain with the strict definition of low inflammation due to the small number of patients in this subset.

As a secondary analysis, predictors of unacceptable pain with high inflammation were assessed in a univariate model. From 6 months to 5 years follow-up, between 10 and 15% of the patients had unacceptable pain plus high inflammation. Significant baseline predictors of this state at 1 and 2 years were seropositivity, high inflammatory parameters, high DAS28 and severe PROs, but not female sex (additional file 5). Patient characteristics of this subset are shown in additional file 6. At 5 years there were no significant predictors of unacceptable pain with high inflammation (additional file 5).

### Predictors of pain over time

In mixed model analysis, baseline predictors of increased pain over time were higher PGA, HAQ, DAS28, tender joint count in 28 joints (TJC28), ESR and CRP in descending order, with the strongest predictor being PGA (Table 6). For each standard deviation of increase of grip force, estimated pain at baseline and over time decreased. Estimated mean differences at baseline were more pronounced than estimated mean differences over time for all variables. Patients with a higher SJC at baseline had higher estimated pain scores at baseline, but the difference in pain over time was not significant. There were no such associations for anti-CCP and RF seropositivity. Patients with worse PROs and disease activity measures at baseline had greater reductions in pain over time, while patients with higher grip force at baseline had less reduction in pain over time (Table 6). Older patients had less pain at baseline and over time, but were also less likely to experience reduced pain during the follow-up (Table 6).

**Table 6:** Baseline predictors of VAS pain over time (from inclusion to 5 years) in early RA

Variable	Estimated mean difference at baseline per SD (95% CI)	Estimated mean difference over time per SD (95% CI)	Difference in change/month per SD (95% CI)
RF seropositivity	3.03 (− 2.31, 8.37)	3.27 (− 1.51, 8.04)	0.01 (− 0.12, 0.15)
Anti-CCP seropositivity	2.99 (− 2.73, 8.71)	2.37 (− 2.78, 7.52)	− 0.03 (− 0.17, 0.10)
HAQ	9.74 (7.32, 12.16)	7.37 (5.24, 9.50)	− 0.13 (− 0.19, − 0.07)
VAS PGA	11.19 (8.85, 13.52)	8.88 (6.85, 10.91)	− 0.13 (− 0.19, − 0.06)
Grip force	− 5.94 (− 8.69, − 3.19)	− 4.64 (− 7.11, − 2.18)	0.07 (0, 0.13)
DAS28	9.62 (7.18, 12.06)	6.80 (4.65, 8.96)	− 0.15 (− 0.22, − 0.09)
SJC28	3.44 (0.85, 6.03)	1.11 (− 1.21, 3.43)	− 0.12 (− 0.19, − 0.06)
TJC28	5.97 (3.42, 8.52)	3.96 (1.69, 6.23)	− 0.11 (− 0.18, − 0.05)
ESR	4.40 (1.82, 6.98)	2.66 (0.35, 4.97)	− 0.10 (− 0.16, − 0.03)
CRP	4.36 (1.80, 6.92)	2.29 (0, 4.58)	− 0.11 (− 0.17, − 0.05)
Age	− 4.48 (− 7.06, − 1.90)	− 3.08 (− 5.38, − 0.78)	0.08 (0.01, 0.14)

Legend: Mixed model analysis, using all VAS pain values at inclusion and at follow-ups after 6 months and 1, 2 and 5 years. *SD* standard deviation, *CI* confidence interval, *RF* rheumatoid factor, *Anti-CCP* anti-cyclic citrullinated peptide, *HAQ* health assessment questionnaire, *VAS* visual analogue scale, *PGA* patient global assessment, *DAS28* disease activity score in 28 joints, *SJC28* swollen joint count in 28 joints, *TJC28* tender joint count in 28 joints, *ESR* erythrocyte sedimentation rate, *CRP* C-reactive protein

## Discussion

In this study, we found that approximately one third of patients with RA have unacceptable pain up to 5 years after diagnosis and that nearly two thirds of these patients have pain despite low inflammatory activity. Pain improvement was only significant between inclusion and the 6 month-visit, and average pain was thereafter essentially unchanged over time. Several baseline variables, including PROs (i.e., VAS pain, VAS PGA and HAQ), and clinical outcomes like low SJC and low grip force, as well as female sex and low age, predicted unacceptable pain. Out of these associations, those with VAS for pain and PGA were consistently significant over time, and both low SJC and high VAS PGA remained significantly associated with unacceptable pain in the multivariate analyses at 5 years. Unacceptable pain with low inflammatory activity was also predicted by low baseline inflammatory parameters and anti-CCP negativity. In mixed model analysis, high baseline PGA VAS, HAQ and DAS28 were particularly associated with increased pain at baseline, but also with greater improvement in pain over time.

Several recent studies have reported that although pain outcome has improved in more recent years since the introduction of more extensive anti-rheumatic treatment and biologic therapy, there is still a group of patients with persistent pain [3, 4, 7]. These findings are further strengthened by our results where more than 30% of the patients had unacceptable pain after 5 years. This points to substantial unmet needs regarding pain management beyond anti-rheumatic treatment and highlights the importance of improved management of these patients. Such pain management might include encouraging patients to engage in physical activity [29], pain-coping strategies [30], and pain-modulating treatment, with for example serotonin and noradrenaline reuptake inhibitors [31]. Treating relevant comorbidities, such as sleep disorders [32] and mental illness [33], when applicable, might also improve pain outcome.

Worse baseline PROs have previously been associated with increased pain levels later during the disease course [7]. For example, in a recent study, RA patients with features of neuropathic pain also had higher self-reported global disease activity, disability, and TJC [5]. In the present study, PROs were the strongest predictors of increased pain over time. Other predictors of unacceptable pain in this study were low age and female sex, both previously reported as risk factors for pain [3, 5, 7, 34]. For instance, female sex has been reported to be a risk factor for chronic widespread pain in patients with RA [5, 34]. In another study, women had significantly higher pain scores than men only at lower disease activity levels [35], suggesting that pain differences by sex might not be present in patients with more active disease. Moreover, in a study investigating pain predictors one year after treatment initiation, pain (derived from the Short-Form 36 questionnaire) was actually associated with male sex and higher age in patients receiving biologic DMARDs [36], suggesting that female sex is a risk factor for increased pain mainly in certain groups, e.g. in patients with low disease activity. The stronger association in the present study between female sex and unacceptable pain plus low inflammation at 2 years, as compared to unacceptable pain plus high inflammation could further support this pattern.

Discrepancies between PROs and objective disease activity measures regarding their importance for pain have been reported earlier [7, 37]. Furthermore, in a cross-sectional study investigating factors associated with non-nociceptive pain, a negative association with anti-CCP positivity was found [5]. In another study, predictors of satisfactory improvement in pain were reported to be anti-CCP positivity and symmetric arthritis [38]. These studies are in line with our results, where approximately 20% of patients had unacceptable pain despite low inflammatory activity during the follow-up. These patients also had lower inflammatory parameters at baseline, and were more likely to be anti-CCP negative, strengthening the concept that there is a group of patients with RA with low disease activity, e.g. low SJC and low laboratory markers of inflammation, as well as anti-CCP negativity, that is more likely to experience a disease course characterized by high pain levels and disability. The current results suggest that patients with anti-CCP antibodies, and more severe clinical disease, respond better to treatment, resulting in reduced long-term pain.

The uncoupling between pain and inflammation supports that non-inflammatory mechanisms contribute to pain in RA. This concept is supported by the association between unacceptable pain and low swollen joint count found in the present study. Furthermore, the prevalence of concomitant fibromyalgia in RA has been reported to be up to 25% [39], as compared to 2% in the general population [40], and in one report, investigating neuropathic pain in patients with RA initiating or escalating anti-rheumatic therapy, 23% and 12% of the patients were diagnosed with possible neuropathic pain, and probable neuropathic pain, respectively [41]. In the latter group, significantly more patients fulfilled the classification criteria for fibromyalgia. These results all indicate that pain in RA is multifactorial and might include central pain mechanisms, encompassing an increased risk of secondary fibromyalgia and persistent nociplastic pain, presumably sharing common mechanisms of disease development, such as central sensitisation. Health care workers should be aware of the uncoupling between pain and inflammation, and in cases of severe pain despite inflammation control consider initiation of targeted multimodal pain interventions.

Some limitations of the present study should also be noted. Data on comorbidities before RA diagnosis were not available, and therefore the effects of other prevalent diagnoses affecting pain, e.g. concomitant fibromyalgia, depression and osteoarthritis, on the results cannot be excluded. Misclassification of individuals with primary fibromyalgia as RA in this study is however unlikely, as even patients with unacceptable pain and low inflammation at follow-up had active disease at inclusion, with median SJCs above 6, and all patients fulfilled the 1987 RA classification criteria at inclusion. The small number of patients was another limitation of the study, as it affects the statistical power, especially for the analyses of unacceptable pain with low inflammation. It would also have been interesting to investigate predictors of unacceptable pain with the strict definition of low inflammation, but the number of patients in this subset was too small. This would be of interest to study in a larger cohort. The data-driven, hypothesis-free approach for selecting covariates for the multivariate models may not be optimal. Other methods, e.g. selection of covariates based on a priori hypothesis, may be useful, in particular in larger samples. Moreover, the majority of patients were included before the practice of treat to target was implemented and before early treatment with biologic DMARDs was standard of care in severe cases, and the results of this study might therefore not be fully applicable to patients diagnosed in the more recent period. However, studies of more recent cohorts demonstrate that pain remains a major problem in patients with RA [4–6, 8]. Finally, pain in RA may also be related to joint damage, which was not analysed in detail in this study, but would be interesting to examine in the future. Yet our result points mainly towards a negative association between pain and baseline presence of erosions.

The strengths of this study include a systematic longitudinal follow-up of patients from a defined period of time and a defined catchment area. Therefore, there should not be a major risk of selection bias, and the results could be generalized to patients with RA seen in clinical practice. Furthermore, all joint assessments were performed by the same rheumatologist for all patients, using a structured protocol. Finally, the definition of unacceptable pain is a common and validated measure, which makes it possible to compare the results of this study to other reports.

## Conclusion

More than one third of patients with new-onset RA suffer from long-lasting unacceptable pain, and the majority of these patients have pain despite the low inflammatory activity. This indicates large unmet needs in RA pain management and highlights that non-inflammatory mechanisms contribute substantially to the pain spectrum. Worse PROs at inclusion, i.e. HAQ, VAS pain and VAS PGA, were associated with unacceptable pain and with a greater burden of pain over time. Furthermore, non-inflammatory long-lasting pain appears to be a greater problem in anti-CCP-negative patients. Future studies should investigate how to improve pain management for such patients, for example by encouraging more effective coping strategies. Patients with less inflammatory disease at baseline, in the form of low swollen joint count, had an increased risk of unacceptable pain at 5 years. This may be explained by the positive effects of treatment on pain related to inflammation.

## Supplementary Information

**Additional file 1:.** Baseline characteristics in early RA patients with or without unacceptable pain at follow-ups.

**Additional file 2:.** Sensitivity analysis – baseline predictors of unacceptable pain in early RA.

**Additional file 3:.** Baseline characteristics in patients with or without unacceptable pain and low inflammation at follow-ups.

**Additional file 4:.** Sensitivity analysis – baseline predictors of unacceptable pain with low inflammation in early RA.

**Additional file 5:.** Baseline predictors of unacceptable pain and high inflammation – 6 months, 1, 2 and 5 years after diagnosis.

**Additional file 6:.** Baseline characteristics in patients with or without unacceptable pain and high inflammation at follow-ups.

## Data Availability

The data underlying this article will be shared on reasonable request to the corresponding author.

## Abbreviations

RA: Rheumatoid arthritis
DMARD: Disease-modifying anti-rheumatic drug
PRO: Patient-reported outcome
VAS: Visual analogue scale
PGA: Patient global assessment
HAQ: Health assessment questionnaire
DAS28: Disease activity score in 28 joints
ESR: Erythrocyte sedimentation rate
CRP: C-reactive protein
RF: Rheumatoid factor
Anti-CCP: Anti-cyclic citrullinated peptide
SJC28: Swollen joint count in 28 joints
OR: Odds ratio
CI: Confidence interval
TJC28: Tender joint count in 28 joints
