# Supplementary material for: Predictors of unacceptable pain with and without low inflammation over 5 years in early rheumatoid arthritis—an inception cohort study
Source: Arthritis Res Ther. 2021 Jun 14;23:169. doi: 10.1186/s13075-021-02550-7 (PMC8201925; doi:10.1186/s13075-021-02550-7)
Supplement: Supplementary file 1 — Additional file 1:. Baseline characteristics in early RA patients with or without unacceptable pain at follow-ups. [file 13075_2021_2550_MOESM1_ESM.docx]

**Additional file 1.**

Title: Baseline characteristics in early RA patients with or without unacceptable pain at follow-ups

| Characteristic | VAS pain>40 after 6 months | | VAS pain>40 after  1 year | | | VAS pain>40 after  2 years | | VAS pain>40 after  5 years | |
| --- | --- | --- | --- | --- | --- | --- | --- | --- | --- |
|  | **Present** | **Absent** | | **Present** | **Absent** | **Present** | **Absent** | **Present** | **Absent** |
| N (%) | 80 (37.7) | 132 (62.3) | | 66 (30.1) | 153 (69.9) | 71 (34.1) | 137 (65.9) | 61 (34.1) | 118 (65.9) |
| Female, n (%) | 56 (70.0) | 94 (71.2) | | 46 (69.7) | 109 (71.2) | 58 (81.7) | 88 (64.2) | 45 (73.8) | 82 (69.5) |
| Age, mean (SD), years | 60.4 (13.6) | 64.0 (12.9) | | 60.1 (13.7) | 63.2 (13.5) | 56.7 (15.9) | 61.8 (14.0) | 60.0 (13.0) | 58.8 (15.3) |
| Symptom duration, months | 7.0 (5.0–10.0) | 7.0 (4.3–10.0) | | 8.0 (6.0–10.3) | 7.0 (5.0–10.0) | 7.0 (5.0–10.0) | 7.0 (5.0–10.0) | 8.0 (6.0–10.0) | 7.0 (5.0–9.0) |
| RF positive, n (%) | 53 (66.3) | 74 (56.1) | | 41 (62.1) | 94 (61.4) | 46 (64.8) | 79 (57.7) | 38 (62.3) | 77 (65.3) |
| Anti-CCP positive, n/N (%) | 42/70 (60.0) | 64/115 (55.7) | | 40/61 (65.6) | 69/128 (53.9) | 36/62 (58.1) | 66/118 (55.9) | 30/55 (54.5) | 61/100 (61.0) |
| Prednisolone, n (%) | 29 (36.3) | 55 (41.7) | | 29 (43.9) | 55 (35.9) | 32 (45.1) | 46 (33.6) | 24 (39.3) | 40 (33.9) |
| Methotrexate, n (%) | 41 (51.1) | 73 (55.3) | | 33 (50.0) | 82 (53.6) | 37 (52.1) | 74 (54.0) | 30 (49.1) | 67 (56.8) |
| No DMARD, n (%) | 12 (15.0) | 24 (18.2) | | 9 (13.6) | 29 (19.0) | 16 (22.5) | 19 (13.9) | 5 (8.2) | 22 (18.6) |
| Erosion, n (%) | 8 (10.0) | 23 (17.4) | | 7 (10.6) | 27 (17.6) | 6 (8.5) | 25 (18.2) | 8 (13.1) | 23 (19.5) |
| Body Mass Index, mean (SD) | 25.9 (4.6)^a^ | 25.2 (4.1)^b^ | | 25.5 (5.1)^c^ | 25.4 (3.8)^d^ | 25.5 (4.6)^e^ | 25.6 (4.0)^f^ | 25.2 (3.9)^g^ | 25.4 (4.0)^h^ |
| Current smoking, n/N (%) | 22/60 (36.7) | 29/91 (31.9) | | 20/49 (40.8) | 33/106 (31.1) | 14/51 (27.5) | 33/96 (34.4) | 17/47 (36.2) | 22/76 (28.9) |
| Grip force, % of expected, mean (SD) | 36.2 (28.1)^i^ | 40.7 (24.0)^j^ | | 33.8 (22.6)^k^ | 41.0 (26.1)^l^ | 33.8 (24.8)^m^ | 41.7 (25.4)^n^ | 34.2 (24.9)^o^ | 40.4 (26.3)^p^ |
| VAS pain, mean (SD) | 48.1 (27.8) | 37.5 (25.6) | | 51.1 (23.6) | 37.4 (27.0) | 48.1 (23.7) | 36.7 (27.4) | 46.4 (24.4) | 37.5 (27.5) |
| DAS28, mean (SD) | 5.0 (1.3) | 4.8 (1.4) | | 4.9 (1.3) | 4.6 (1.5) | 4.9 (1.5) | 4.5 (1.4) | 4.7 (1.2) | 4.6 (1.5) |
| SJC28 | 7.0 (5.0–11.0) | 7.0 (4.0–10.8) | | 7.0 (4.0–10.0) | 7.0 (5.0–11.0) | 7.0 (4.0–11-0) | 7.0 (5.0–11.0) | 6.0 (4.0–8.0) | 7.5 (4.8–12.0) |
| TJC28 | 5.0 (2.0–9.8) | 4.0 (1.0–8.8) | | 6.0 (3.0–11.0) | 3.0 (1.0–8.5) | 5.0 (3.0–10-0) | 3.0 (1.0–10.0) | 5.0 (2.0–8.0) | 4.0 (1.0–10.3) |
| HAQ | 0.9 (0.5–1.5) | 0.8 (0.4–1.1) | | 1.0 (0.6–1.4) | 0.8 (0.3–1.1) | 1.0 (0.5–1.5) | 0.8 (0.3–1.1) | 0.8 (0.4–1.1) | 0.8 (0.3–1.3) |
| CRP (mg/l) | <9 (<9–22.0) | 10 (<9–29.5) | | <9 (<9–22.0) | 10.0 (<9–30.0) | <9 (<9–32.0) | 9.0 (<9–24.0) | 9.0 (<9–17.5) | 10.0 (<9–32.5) |
| CRP>9 mg/l, n (%) | 39 (48.8) | 72 (54.5) | | 33 (50.0) | 79 (51.6) | 33 (46.5) | 72 (52.6) | 31 (50.8) | 64 (54.2) |
| ESR (mm/h) | 20.0 (11.5–49.8) | 21.0 (10.0–39.0) | | 19.5 (10.0–44.0) | 21.0 (10.0–41.5) | 19.0 (10.0–44.0) | 21.0 (10.0–43.0) | 19.0 (10.0–31.5) | 24.0 (11.0–47.8) |
| VAS PGA, mean (SD) | 57.2 (26.5) | 39.2 (25.6) | | 50.7 (25.7) | 41.0 (27.0) | 50.5 (24.3) | 39.4 (27.5) | 51.0 (22.8) | 38.8 (27.7) |

Legend: Values are median (interquartile range) unless otherwise indicated. ^a^Data for body mass index in 60 cases. ^b^Data in 90 cases. ^c^Data in 48 cases. ^d^Data in 106 cases. ^e^Data in 51 cases. ^f^Data in 95 cases. ^g^Data in 46 cases. ^h^Data in 76 cases. ^i^Data gor grip force in 80 cases. ^j^Data in 116 cases. ^k^Data for grip force in 55 cases. ^l^Data in 136 cases. ^m^Data in 63 cases. ^n^Data in 123 cases. ^o^Data in 59 cases. ^p^Data in 105 cases.
VAS: visual analogue scale; SD: standard deviation; RF: rheumatoid factor; Anti-CCP: anti-cyclic citrullinated peptide; DMARD: disease-modifying anti-rheumatic drug; DAS28: disease activity score in 28 joints; SJC28: swollen joint count in 28 joints; TJC28: tender joint count in 28 joints; HAQ: health assessment questionnaire; CRP: C-reactive protein; ESR: erythrocyte sedimentation rate; PGA: patient global assessment.
