# Supplementary material for: Predictors of unacceptable pain with and without low inflammation over 5 years in early rheumatoid arthritis—an inception cohort study
Source: Arthritis Res Ther. 2021 Jun 14;23:169. doi: 10.1186/s13075-021-02550-7 (PMC8201925; doi:10.1186/s13075-021-02550-7)
Supplement: Supplementary file 2 — Additional file 2:. Sensitivity analysis – baseline predictors of unacceptable pain in early RA. [file 13075_2021_2550_MOESM2_ESM.docx]

**Additional file 2.**

Title: Sensitivity analysis – baseline predictors of unacceptable pain in early RA

| Variable | Odds ratio | 95% CI | P-value |
| --- | --- | --- | --- |
|  | **2 years after inclusion** |  |  |
| VAS pain | 1.62 | 1.17–2.25 | <0.01 |
| Female sex | 2.56 | 1.22–5.34 | 0.01 |
| Age | 0.78 | 0.58–1.05 | 0.10 |
| Erosion | 0.54 | 0.20–1.43 | 0.22 |
|  | **5 years after inclusion** | | |
| PGA | 1.93 | 1.34–2.78 | <0.01 |
| SJC28 | 0.69 | 0.47–1.00 | 0.05 |
|  |  |  |  |

Legend: Multivariate logistic regression analysis, adjusted for *year of inclusion* and *practice* (university vs private). Odds ratios are calculated per standard deviation for continuous variables. Unacceptable pain: VAS pain>40. CI: confidence interval; VAS: visual analogue scale; PGA: patient global assessment; SJC28: swollen joint count in 28 joint; PGA: patient global assessment.
