# Supplementary material for: Predictors of unacceptable pain with and without low inflammation over 5 years in early rheumatoid arthritis—an inception cohort study
Source: Arthritis Res Ther. 2021 Jun 14;23:169. doi: 10.1186/s13075-021-02550-7 (PMC8201925; doi:10.1186/s13075-021-02550-7)
Supplement: Supplementary file 3 — Additional file 3:. Baseline characteristics in patients with or without unacceptable pain and low inflammation at follow-ups. [file 13075_2021_2550_MOESM3_ESM.docx]

**Additional file 3.**

| Characteristic | VAS pain>40 and CRP<10 after 6 months | | VAS pain>40 and CRP<10  after 1 year | | | VAS pain>40 and CRP<10  after 2 years | | VAS pain>40 and CRP<10  after 5 years | |
| --- | --- | --- | --- | --- | --- | --- | --- | --- | --- |
|  | **Present** | **Absent** | | **Present** | **Absent** | **Present** | **Absent** | **Present** | **Absent** |
| N (%) | 45 (21.3) | 166 (78.7) | | 40 (18.3) | 178 (81.7) | 41 (19.7) | 167 (80.3) | 40 (22.5) | 138 (77.5) |
| Female, n (%) | 33 (73.3) | 117 (70.5) | | 27 (67.5) | 127 (71.3) | 35 (85.4) | 111 (66.5) | 32 (80.0) | 94 (68.1) |
| Age, mean (SD), years | 59.9 (14.6) | 63.3 (12.9) | | 56.1 (14.0) | 63.6 (13.2) | 53.7 (17.5) | 61.6 (13.7) | 58.8 (13.4) | 59.3 (14.9) |
| Symptom duration, months | 8.0 (6.0–10.0) | 7.0 (5.0–10.0) | | 8.5 (6.3–10.0) | 7.0 (5.0–10.0) | 7.0 (5.0–9.0) | 7.0 (5.0–10.0) | 8.0 (6.0–10.0) | 7.0 (5.0–10.0) |
| RF positive, n (%) | 24 (53.3) | 102 (61.4) | | 22 (55.0) | 112 (62.9) | 23 (56.1) | 102 (61.1) | 21 (52.5) | 93 (67.4) |
| Anti-CCP positive, n/N (%) | 21/40 (52.5) | 84/144 (58.3) | | 21/47 (56.8) | 88/151 (58.3) | 16/34 (47.1) | 86/146 (58.9) | 16/36 (44.4) | 75/118 (63.6) |
| Prednisolone, n (%) | 15 (33.3) | 69 (41.6) | | 16 (40.0) | 68 (38.2) | 18 (43.9) | 60 (35.9) | 15 (37.5) | 49 (35.5) |
| Methotrexate, n (%) | 22 (48.9) | 92 (55.4) | | 19 (47.5) | 96 (53.9) | 20 (48.8) | 91 (54.5) | 21 (52.2) | 75 (54.3) |
| No DMARD, n (%) | 7 (15.6) | 29 (17.5) | | 6 (15.0) | 32 (18.0) | 12 (29.3) | 23 (13.8) | 5 (12.5) | 22 (15.9) |
| Erosion, n (%) | 1 (2.2) | 29 (17.5) | | 4 (10.0) | 30 (16.9) | 2 (4.9) | 29 (17.4) | 5 (12.5) | 26 (18.8) |
| Body Mass Index, mean (SD) | 26.3 (4.9)^a^ | 25.2 (4.0)^b^ | | 25.1 (4.7)^c^ | 25.5 (4.2)^d^ | 25.4 (4.4)^e^ | 25.6 (4.2)^f^ | 25.3 (3.9)^g^ | 25.4 (4.0)^h^ |
| Current smoking, n/N (%) | 7/32 (21.9) | 43/118 (36.4) | | 10/28 (35.7) | 43/126 (34.1) | 5/27 (18.5) | 42/120 (35.0) | 8/29 (27.6) | 31/94 (33.0) |
| Grip force, % of expected, mean (SD) | 38.2 (26.8)^i^ | 39.1 (25.3)^j^ | | 36.4 (23.3)^k^ | 39.4 (25.8)^l^ | 35.3 (22.0)^m^ | 40.0 (26.2)^n^ | 37.1 (23.1)^o^ | 40.2 (26.7)^p^ |
| VAS pain, mean (SD) | 46.8 (27.3) | 40.0 (26.7) | | 49.0 (24.3) | 39.7 (27.0) | 45.6 (23.2) | 39.3 (27.4) | 46.8 (23.4) | 38.9 (27.5) |
| DAS28, mean (SD) | 4.7 (1.3) | 4.7 (1.4) | | 4.6 (1.3) | 4.7 (1.5) | 4.6 (1.4) | 4.6 (1.4) | 4.6 (1.2) | 4.6 (1.4) |
| SJC28 | 7.0 (4.5–10.0) | 7.0 (5.0–11.0) | | 6.0 (4.0–9.8) | 7.0 (5.0–11.0) | 7.0 (4.0–11.0) | 7.0 (5.0­–11.0) | 6.0 (4.0–8.0) | 7.0 (4.8–12.0) |
| TJC28 | 5.0 (2.0–12.5) | 4.0 (1.0–8.3) | | 7.0 (3.0–11.0) | 4.0 (1.0–9.0) | 6.0 (3.5–10.5) | 4.0 (1.0–10.0) | 5.0 (2.0–8.8) | 4.0 (1.0–9.0) |
| HAQ | 0.8 (0.3–1.2) | 0.9 (0.5–1.3) | | 0.8 (0.6–1.3) | 0.9 (0.3–1.3) | 0.9 (0.4–1.4) | 0.8 (0.4–1.3) | 0.75 (0.41–1.10) | 0.75 (0.25–1.25) |
| CRP (mg/l) | <9 (<9–11.0) | 10 (<9–34.0) | | <9 (<9–9.0) | 10.0 (<9–34.3) | < 9 (< 9–10.5) | 10.0 (< 9–30.0) | < 9 (< 9–14.0) | 9.5 (< 9–33.3) |
| CRP>9 mg/l, n (%) | 15 (33.3) | 95 (57.2) | | 12 (30.0) | 100 (56.2) | 15 (36.6) | 90 (53.9) | 20 (50.0) | 74 (53.6) |
| ESR (mm/h) | 16.0 (8.5–27.5) | 23.5 (11.0–45.0) | | 16.0 (9.0–22.0) | 23.0 (11.0–44.0) | 14.0 (9.5–27.0) | 22.0 (11.0–45.0) | 18.0 (7.8–30.0) | 23.0 (11.0–46.3) |
| VAS PGA, mean (SD) | 46.7 (25.0) | 43.1 (27.4) | | 46.4 (25.8) | 43.2 (27.2) | 50.0 (24.0-64.0) | 42.0 (27.5) | 49.8 (22.1) | 41.0 (27.8) |

Title: Baseline characteristics in patients with or without unacceptable pain and low inflammation at follow-ups

Legend: Values are median (interquartile range) unless otherwise indicated. ^a^Data for body mass index in 32 cases. ^b^Data in 117 cases. ^c^Data in 28 cases. ^d^Data in 125 cases. ^e^Data in 27 cases. ^f^Data in 119 cases. ^g^Data in 29 cases. ^h^Data in 93 cases. ^i^Data for grip force in 42 cases. ^j^Data in 142 cases. ^k^Data in 37 cases. ^l^Data in 153 cases. ^m^Data in 37 cases. ^n^Data in 149 cases. ^o^Data in 39 cases. ^p^Data in 124 cases.
VAS: visual analogue scale; CRP: C-reactive protein; SD: standard deviation; RF: rheumatoid factor; Anti-CCP: anti-cyclic citrullinated peptide; DMARD: disease-modifying anti-rheumatic drug; DAS28: disease activity score in 28 joints; SJC28: swollen joint count in 28 joints; TJC28: tender joint count in 28 joints; HAQ: health assessment questionnaire; ESR: erythrocyte sedimentation rate; PGA: patient global assessment.
