# Supplementary material for: Predictors of unacceptable pain with and without low inflammation over 5 years in early rheumatoid arthritis—an inception cohort study
Source: Arthritis Res Ther. 2021 Jun 14;23:169. doi: 10.1186/s13075-021-02550-7 (PMC8201925; doi:10.1186/s13075-021-02550-7)
Supplement: Supplementary file 4 — Additional file 4:. Sensitivity analysis – baseline predictors of unacceptable pain with low inflammation in early RA. [file 13075_2021_2550_MOESM4_ESM.docx]

**Additional file 4.**

Title: Sensitivity analysis – baseline predictors of unacceptable pain with low inflammation in early RA

| Variable | Odds ratio | 95% CI | P-value |
| --- | --- | --- | --- |
|  | **1 year after inclusion** |  |  |
| VAS pain | 1.69 | 1.13–2.50 | 0.01 |
| ESR | 0.56 | 0.34–0.93 | 0.02 |
| Age | 0.69 | 0.49–0.99 | 0.04 |
|  | **2 years after inclusion** | | |
| Female sex | 2.37 | 0.91–6.16 | 0.08 |
| Age | 0.72 | 0.51–1.02 | 0.06 |
| Erosion | 0.31 | 0.07–1.43 | 0.13 |
| CRP<9 mg/l | 1.00 (reference) | - | - |
| CRP 9–27.4 mg/l | 0.77 | 0.31–1.92 | 0.58 |
| CRP≥27.5 mg/l | 0.45 | 0.15–1.36 | 0.16 |
|  | **5 years after inlcusion** |  |  |
| Anti-CCP seropositivity | 0.47 | 0.21–1.05 | 0.07 |
| SJC28 | 0.67 | 0.41–1.11 | 0.12 |
| PGA | 1.58 | 1.04–2.40 | 0.03 |
|  |  |  |  |

Legend: Multivariate logistic regression analysis, adjusted for *year of inclusion* and *practice* (university vs private). Odds ratios are calculated per standard deviation for continuous variables. Unacceptable pain: VAS pain>40. Low inflammation: CRP<10 mg/l. CI: confidence interval; VAS: visual analogue scale; ESR: erythrocyte sedimentation rate; CRP: C-reactive protein; Anti-CCP: anti-cyclic citrullinated peptide; SJC28: swollen joint count in 28 joint; PGA: patient global assessment.
