# Supplementary material for: Predictors of unacceptable pain with and without low inflammation over 5 years in early rheumatoid arthritis—an inception cohort study
Source: Arthritis Res Ther. 2021 Jun 14;23:169. doi: 10.1186/s13075-021-02550-7 (PMC8201925; doi:10.1186/s13075-021-02550-7)
Supplement: Supplementary file 5 — Additional file 5:. Baseline predictors of unacceptable pain and high inflammation – 6 months, 1, 2 and 5 years after diagnosis. [file 13075_2021_2550_MOESM5_ESM.docx]

**Additional file 5.**

Title: Baseline predictors of unacceptable pain and high inflammation – 6 months, 1, 2 and 5 years after diagnosis.

|  | 6 months | 1 year | 2 years | 5 years |
| --- | --- | --- | --- | --- |
| Variable | **Odds ratio  (95% CI)** | **Odds ratio  (95% CI)** | **Odds ratio  (95% CI)** | **Odds ratio  (95% CI)** |
| Female sex | 0.74 (0.34–1.60) | 1.15 (0.46–2.88) | 1.47 (0.60–3.63) | 0.63 (0.25–1.63) |
| RF seropositivity | **3.94 (1.56–9.96)** | 1.82 (0.73–4.53) | **2.45 (1.00–6.00)** | 2.63 (0.84–8.19) |
| Anti-CCP seropositivity | 1.94 (0.84–4.52) | **3.12 (1.12–8.77)** | 2.13 (0.89–5.14) | 2.11 (0.72–6.19) |
| Erosion | 1.66 (0.65–4.25) | 0.68 (0.19–2.40) | 0.86 (0.28–2.66) | 0.77 (0.21–2.79) |
| Age | 1.04 (0.72–1.51) | 1.42 (0.89–2.52) | 1.05 (0.71–1.55) | 1.30 (0.79–2.13) |
| Symptom duration | 0.94 (0.65–1.36) | 0.96 (0.63–1.45) | 1.08 (0.73–1.59) | 1.26 (0.79–2.01) |
| Body Mass Index | 0.99 (0.66–1.49) | 1.20 (0.77–1.86) | 1.02 (0.67–1.58) | 0.80 (0.93–1.62) |
| Current smoking | **2.87 (1.24–6.64)** | 1.90 (0.75–4.82) | 1.34 (0.54–3.34) | 2.50 (0.91–6.91) |
| Grip force | 0.74 (0.46–1.17) | 0.56 (0.29–1.05) | 0.67 (0.41–1.10) | 0.96 (0.60–1.54) |
| VAS pain | **1.45 (1.01–2.10)** | **1.74 (1.14–2.67)** | **1.60 (1.08–2.38)** | 1.23 (0.78–1.94) |
| DAS28 | **1.89 (1.26–2.84)** | **1.73 (1.12–2.69)** | **1.57 (1.05–2.35)** | 1.08 (0.68–1.70) |
| SJC28 | 1.23 (0.87–1.73) | 0.95 (0.63–1.44) | 1.05 (0.72–1.51) | 0.85 (0.53–1.36) |
| TJC28 | 1.09 (0.77–1.54) | 1.12 (0.76–1.65) | 1.02 (0.70–1.48) | 0.87 (0.52–1.45) |
| HAQ | **1.55 (1.08–2.21)** | **1.90 (1.27–2.84)** | **1.89 (1.29–2.76)** | 0.99 (0.63–1.55) |
| CRP<9 mg/l | 1.00 (reference) | 1.00 (reference) | 1.00 (reference) | 1.00 (reference) |
| CRP 9–27.4 mg/l | 1.74 (0.69–4.40) | **3.23 (1.01–10.39)** | 0.76 (0.25–2.28) | 0.82 (0.26–2.56) |
| CRP 27.5–174 mg/l | **2.98 (1.23–7.04)** | **6.41 (2.15–19.12)** | **2.66 (1.11–6.38)** | 1.17 (0.40–3.46) |
| ESR (mm/h) | **1.71 (1.24–2.37)** | **1.60 (1.12–2.29)** | **1.78 (1.26–2.51)** | 0.96 (0.60–1.52) |
| VAS PGA | **1.85 (1.26–2.74)** | **1.81 (1.17–2.79)** | **1.63 (1.09–2.43)** | 1.55 (0.97–2.47) |

Legend: Univariate logistic regression analysis. Odds ratios are calculated per standard deviation for continuous variables. Unacceptable pain: VAS pain>40. High inflammation: CRP≥10 mg/l. Values in bold indicate statistical significance with p-values <0.05. CI: confidence interval; RF: rheumatoid factor; Anti-CCP: anti-cyclic citrullinated peptide; VAS: visual analogue scale; DAS28: disease activity score in 28 joints; SJC28: swollen joint count in 28 joints; TJC28: tender joint count in 28 joints; HAQ: health assessment questionnaire; CRP: C-reactive protein; ESR: erythrocyte sedimentation rate; PGA: patient global assessment.
