# Supplementary material for: Predictors of unacceptable pain with and without low inflammation over 5 years in early rheumatoid arthritis—an inception cohort study
Source: Arthritis Res Ther. 2021 Jun 14;23:169. doi: 10.1186/s13075-021-02550-7 (PMC8201925; doi:10.1186/s13075-021-02550-7)
Supplement: Supplementary file 6 — Additional file 6:. Baseline characteristics in patients with or without unacceptable pain and high inflammation at follow-ups. [file 13075_2021_2550_MOESM6_ESM.docx]

**Additional file 6.**

| Characteristic | VAS pain>40 and CRP≥10 after 6 months | | VAS pain>40 and CRP≥10 after  1 year | | | VAS pain>40 and CRP≥10 after  2 years | | VAS pain>40 and CRP≥10 after  5 years | |
| --- | --- | --- | --- | --- | --- | --- | --- | --- | --- |
|  | **Present** | **Absent** | | **Present** | **Absent** | **Present** | **Absent** | **Present** | **Absent** |
| N (%) | 35 (16.6) | 176 (83.4) | | 26 (11.9) | 192 (88.1) | 30 (14.4) | 178 (85.6) | 21 (11.8) | 157 (88.2) |
| Female, n (%) | 23 (65.7) | 127 (72.2) | | 19 (73.1) | 135 (70.3) | 23 (76.7) | 123 (69.1) | 13 (61.9) | 113 (72.0) |
| Age, mean (SD), years | 61.1 (12.7) | 63.0 (13.4) | | 65.6 (11.4) | 61.6 (14.0) | 62.6 (12.8) | 61.8 (14.2) | 61.9 (11.3) | 61.0 (1419) |
| Symptom duration, months | 7.0 (5.0–9.0) | 7.0 (5.0–10.0) | | 7.0 (4.8–11.0) | 7.0 (5.0–10.0) | 8.0 (4.0–11.0) | 7.0 (5.0–10.0) | 10.0 (5.0–10.5) | 7.0 (5.0–9.5) |
| RF positive, n (%) | 29 (82.9) | 97 (55.1) | | 19 (73.1) | 115 (59.9) | 23 (76.7) | 102 (57.3) | 17 (81.0) | 97 (61.8) |
| Anti-CCP positive, n/N (%) | 21/30 (70.0) | 84/154 (54.5) | | 19/24 (79.2) | 90/164 (54.9) | 20/28 (71.4) | 82/152 (53.9) | 14/19 (73.7) | 77/135 (57.0) |
| Prednisolone, n (%) | 14 (40.0) | 70 (39.8) | | 13 (50.0) | 71 (37.0) | 18 (43.9) | 64 (36.0) | 9 (42.9) | 55 (35.0) |
| Methotrexate, n (%) | 19 (54.3) | 95 (42.0) | | 14 (53.8) | 101 (52.6) | 17 (56.7) | 94 (52.8) | 9 (42.9) | 87 (55.4) |
| No DMARD, n (%) | 5 (14.3) | 31 (17.6) | | 3 (11.5) | 35 (18.2) | 4 (13.3) | 31 (17.4) | 0 (0) | 27 (17.2) |
| Erosion, n (%) | 7 (20.0) | 23 (13.1) | | 3 (11.5) | 31 (16.1) | 14 (46.7) | 27 (15.2) | 3 (14.3) | 28 (17.8) |
| Body Mass Index, mean (SD) | 25.4 (3.9)^a^ | 25.4 (4.3)^b^ | | 26.1 (5.7)^c^ | 25.3 (4.0)^d^ | 25.6 (5.0)^e^ | 25.3 (4.1)^f^ | 25.1 (4.2)^g^ | 25.4 (3.9)^h^ |
| Current smoking, n/N (%) | 15/28 (53.6) | 35/122 (28.7) | | 10/21 (47.6) | 43/133 (32.3) | 9/24 (37.5) | 38/123 (30.9) | 9/18 (50.0) | 30/105 (28.6) |
| Grip force, % of expected, mean (SD) | 33.0 (30.1)^i^ | 40.0 (24.7)^j^ | | 28.4 (20.6)^k^ | 39.9 (28.8)^l^ | 31.6 (28.7)^m^ | 40.3 (24.8)^n^ | 38.6 (28.6)^o^ | 39.6 (25.5)^p^ |
| VAS pain, mean (SD) | 49.9 (28.6) | 39.8 (26.3) | | 54.2 (22.5) | 39.7 (26.8) | 51.2 (24.4) | 38.8 (26.7) | 45.6 (26.8) | 40.0 (26.9) |
| DAS28, mean (SD) | 5.4 (1.3) | 4.5 (1.4) | | 5.3 (1.3) | 4.6 (1.4) | 5.4 (1.7) | 4.6 (1.4) | 4.7 (1.3) | 4.6 (1.4) |
| SJC28 | 8.0 (5.0–12.0) | 7.0 (4.3–10.8) | | 8.0 (5.0–10.0) | 7.0 (4.3–11.0) | 7.0 (5.0–12.3) | 7.0 (4.0­–11.0) | 6.0 (4.5–9.5) | 7.0 (4.0–11.0) |
| TJC28 | 5.0 (2.0–8.0) | 4.0 (1.0–9.0) | | 6.0 (3.5–10.5) | 4.0 (1.0–9.0) | 4.5 (2.0–10.5) | 4.0 (1.8–10.3) | 3.0 (2.0–7.0) | 4.0 (1.0–9.0) |
| HAQ | 1.0 (0.8–1.5) | 0.8 (0.4–1.1) | | 1.2 (0.8–1.8) | 0.8 (0.3–1.2) | 1.1 (0.7–1.8) | 0.8 (0.3–1.1) | 0.9 (0.3–1.3) | 0.8 (0.3–1.3) |
| CRP (mg/l) | 18.0 (<9–50.0) | <9 (<9–22.0) | | 25.0 (9–40.0) | <9 (<9–21.0) | 16.0 (<9–48.0) | <9 (<9–21.0) | 9.0 (<9–35.0) | 9.0 (<9–25.5) |
| CRP>9 mg/l, n (%) | 24 (68.6) | 86 (48.9) | | 21 (80.8) | 91 (47.4) | 18 (60.0) | 87 (48.9) | 11 (52.4) | 83 (52.9) |
| ESR (mm/h) | 40.0 (19.0–70.0) | 18.0 (10.0–34.8) | | 32.5 (17.3–75.5) | 19.0 (10.0–37.5) | 32.5 (18.0–79.8) | 18.0 (10.0–36.5) | 21.0 (13.0–43.5) | 22.0 (10.5–43.5) |
| VAS PGA, mean (SD) | 57.2 (26.5) | 41.3 (26.2) | | 57.4 (24.6) | 41.9 (26.7) | 54.1 (24.4) | 41.3 (26.9) | 53.1 (24.6) | 41.6 (26.9) |

Title: Baseline characteristics in patients with or without unacceptable pain and high inflammation at follow-ups

Legend: Values are median (interquartile range) unless otherwise indicated. ^a^Data for body mass index in 28 cases. ^b^Data in 121 cases. ^c^Data in 20 cases. ^d^Data in 133 cases. ^e^Data in 24 cases. ^f^Data in 122 cases. ^g^Data in 17 cases. ^h^Data in 105 cases. ^i^Data for grip force in 27 cases. ^j^Data in 157 cases. ^k^Data in 18 cases. ^l^Data in 172 cases. ^m^Data in 26 cases. ^n^Data in 160 cases. ^o^Data in 20 cases. ^p^Data in 143 cases.
VAS: visual analogue scale; CRP: C-reactive protein; SD: standard deviation; RF: rheumatoid factor; Anti-CCP: anti-cyclic citrullinated peptide; DMARD: disease-modifying anti-rheumatic drug; DAS28: disease activity score in 28 joints; SJC28: swollen joint count in 28 joints; TJC28: tender joint count in 28 joints; HAQ: health assessment questionnaire; ESR: erythrocyte sedimentation rate; PGA: patient global assessment.
